# Meta-Analysis of the Efficacy and Safety of Alendronate Combined with Atorvastatin in the Treatment of Osteoporosis in Diabetes Mellitus

**DOI:** 10.1155/2022/6747469

**Published:** 2022-02-07

**Authors:** Zhencheng Xiong, Ping Yi, Xiangsheng Tang, Li Shu, Chi Zhang

**Affiliations:** ^1^Department of Spine Surgery, China-Japan Friendship Hospital, Beijing 100029, China; ^2^Department of Orthopedics, Peking University International Hospital, Beijing 102206, China; ^3^Biomedical Engineering Department, Peking University, Beijing 100191, China

## Abstract

**Objective:**

Diabetes is a chronic disease caused by defective insulin secretion in the body, resulting in metabolic abnormalities with persistent blood glucose elevation. Osteoporosis is the most common diabetes complication. The aim of this study was to perform a meta-analysis of the effects of alendronate combined with atorvastatin compared with alendronate alone in the treatment of osteoporosis in diabetes mellitus.

**Methods:**

Two researchers independently used PubMed, ScienceDirect, Cochrane Library, Wanfang Data, CNKI, and VIP databases to search for all relevant studies that met the inclusion criteria and used RevMan 5.3 and STATA 16.0 for data analysis.

**Results:**

Fourteen studies that met the inclusion criteria were selected, including 1456 patients. Among the data extracted in this meta-analysis, bone mineral density (BMD) was the primary outcome measurement, while total effective rate, VAS, osteoprotegerin (OPG), bone Gla protein (BGP), bone alkaline phosphatase (BAP), blood P and Ca, and adverse effects were secondary outcome measurements. Our results showed that alendronate combined with atorvastatin is more effective than alendronate alone, with higher BMD, OPG, BGP, and BAP, more significant pain relief, and fewer adverse events.

**Conclusion:**

The results of this meta-analysis indicate that alendronate combined with atorvastatin is a better treatment for osteoporosis in diabetes mellitus, showing more effective and higher BMD and fewer adverse events than alendronate alone.

## 1. Introduction

The prevalence of diabetes mellitus has increased significantly with the aging of populations in recent decades [1]. Diabetes is a chronic disease caused by the defective insulin secretion in the body, resulting in abnormal metabolism with a continuous increase in blood sugar [2]. Diabetes may cause different complications, of which osteoporosis is the most common one [3]. Diabetes causes bone metabolism disorders and reduces bone mineral content, which lead to osteoporosis. If diabetes-caused osteoporosis is left untreated, bone pain will occur, which may lead to disability eventually in severe cases [3]. Alendronate is an aminobisphosphonate that acts as a potent inhibitor of bone resorption [4]. A clinical study showed that 70 mg of alendronate per week was effective in improving bone mineral density (BMD) and reducing bone loss in patients with proximal femur osteoporosis [4]. Atorvastatin is a statin drug widely used to lower cholesterol levels [5]. Statins have been reported to have multiple effects, such as antioxidant properties, inhibition of inflammatory response, and bone metabolism. A nationwide population-based cohort study showed that atorvastatin had a potential protective effect on osteoporosis [5]. In addition, statins directly affect osteoclasts through a mechanism similar to bisphosphonates. Both statins and bisphosphonates exert their effects through the mevalonate pathway. However, alendronate is not highly bioavailable and easily binds to plasma proteins, resulting in a low bone tissue resorption rate and unsatisfactory therapeutic effect [6]. Therefore, combining alendronate with atorvastatin to treat osteoporosis in diabetes mellitus has become a new approach, and it has been addressed in some studies [7–20]. In order to investigate the effect of the alendronate combined with atorvastatin group compared to the alendronate alone group in patients with osteoporosis in diabetes mellitus, we performed this meta-analysis by pooling the relevant studies.

## 2. Materials and Methods

### 2.1. Search Method

In order to obtain all relevant studies to the study topic, two researchers independently searched multiple databases according to the Cochrane Collaboration guidelines, including PubMed (1966 to October 1, 2021), ScienceDirect (1990 to October 1, 2021), Cochrane library (1966 to October 1, 2021), Wanfang Data (1990 to October 1, 2021), CNKI (1990 to October 1, 2021), and Chinese Scientific Journal Database (VIP) (1990 to October 1, 2021). The relevant study search was achieved by using Boolean operators (AND or OR) to link MeSH terms to their corresponding keywords, including “alendronate,” “atorvastatin,” “diabetes mellitus or diabetes,” and “osteoporosis.” The two researchers independently screened all retrieved articles, first on a title-by-title and abstract-by-abstract basis, followed by a detailed reading of the full text, and also looked through the references of the screened articles for potentially compatible studies. The final obtained studies are discussed and integrated. The Preferred Reporting Items for Systematic Reviews and Meta-Analyses (PRISMA) statement is an important reference for this meta-analysis [21].

### 2.2. Study Screening

Screening of all retrieved articles was performed according to the inclusion and exclusion criteria developed for the topics of this meta-analysis. Inclusion criteria included the following: (1) all studies involved a comparison of alendronate combined with atorvastatin versus atorvastatin alone for the treatment of osteoporosis in diabetes mellitus, (2) all included studies were randomized controlled trials (RCTs), and (3) data relevant to the outcome measures could be successfully extracted. Exclusion criteria included the following: (1) studies that were lacking a control group; (2) relevant data for the outcome measures could not be extracted; (3) the type of study was a review, conference abstract, commentary, case report, or letter; and (4) all studies that did not meet the inclusion criteria.

### 2.3. Data Extraction

Two researchers independently complete the extraction of the required data, and then, another researcher summarizes the above data and resolves the divergent data after discussion within the research team. Of the data extracted in this meta-analysis, BMD was the primary outcome measurement, while total effective rate, VAS, osteoprotegerin (OPG), bone Gla protein (BGP), bone alkaline phosphatase (BAP), blood P and Ca, and adverse effects were secondary outcome measurements. The following data were also extracted: first author, year of publication, country/region, study type, drug dose and month of use (experimental group : control group), body mass index (BMI), and gender.

### 2.4. Quality Assessment

The Cochrane Handbook of Systematic Reviews is commonly used to assess the quality of RCTs in meta-analyses [22]. Two researchers used a “risk of bias” table with seven main elements to assess the quality of each included RCT. Depending on the actual content of the study, each element could be judged as high risk of bias, low risk of bias, or one of the unclear risks of bias.

### 2.5. Statistical Analysis

Subgroup analyses were performed according to the period of atorvastatin application, as well as the time of detection of outcome measurements. When included outcome measurements were continuous data, as well as unit differences, we used standardized mean differences (SMD) and 95% confidence intervals (CI) for analysis; risk ratio (RR) and 95% CI were used when dichotomous data were included. Heterogeneity of included studies was assessed by *I*^2^ and was considered low, moderate, and high when *I*^2^ values were 25%, 50%, and 75%, respectively [23]. The magnitude of *I*^2^ determined the choice of the random effects and fixed effects models, with the former executed when *I*^2^ > 50% and *P* < 0.1; otherwise, the latter was executed. We used STATA software version 16.0 and RevMan 5.3 for Windows for statistical analysis of all data. The results of the meta-analysis were considered statistically significant when *P* < 0.05.

## 3. Results

### 3.1. Search Results for Literature

**Figure 1:**
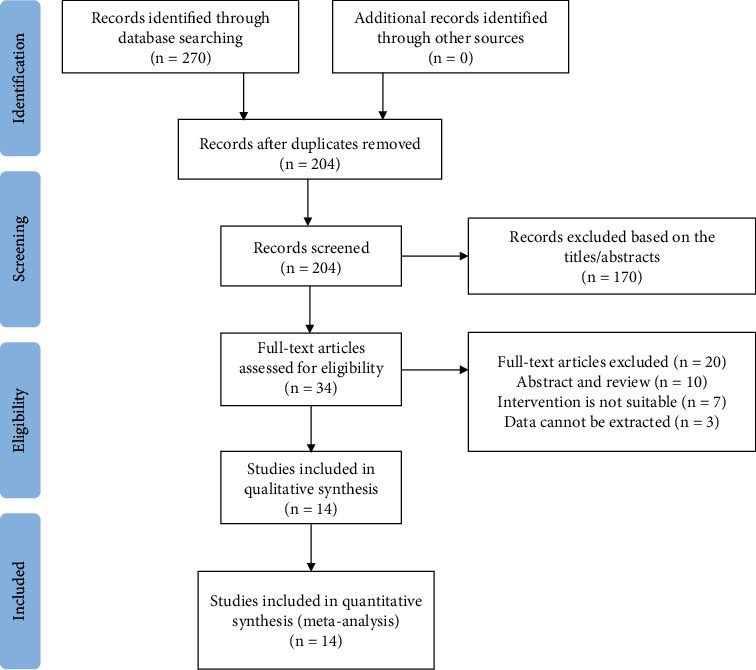
Flow chart of literature search and screening for meta-analysis.

A total of 270 potentially relevant articles were generated based on the search strategy and inclusion and exclusion criteria, including PubMed (*n* = 1), ScienceDirect (*n* = 177), Cochrane Library (*n* = 0), Wanfang Data (*n* = 34), CNKI (*n* = 31), and VIP (*n* = 27). A total of 170 articles were excluded after careful independent screening of titles and abstracts and brief review of the full text by two researchers. The full text of the remaining 34 articles was then evaluated in detail based on the inclusion and exclusion criteria. Finally, the meta-analysis included 14 RCTs (Figure 1) [7–20].

### 3.2. Study Characteristics

A total of 14 RCTs involving 1456 patients, published between 2017 and 2021, were included in this meta-analysis [7–20]. All included studies investigated the effect of the alendronate combined with atorvastatin group compared to the alendronate alone group in patients with osteoporosis in diabetes mellitus. A total of 10 studies have used BMD as the primary outcome measurement [7, 8, 10, 11, 13–18]. BMD was classified into four types depending on the site of measurement, including femoral neck, femoral rotor, forearm, and lumbar spine. Each type of BMD was further divided into 4 subgroups according to the time of detection and the period of application of the intervention. Five studies were also conducted to assess the difference in efficacy between the two groups by serological examination, including OPG, BGP, BAP, blood P, and Ca [15–18, 20]. However, there were also 12 studies that reported adverse effects associated with the application of both groups of interventions, including headache, abdominal pain, nausea, vomiting, and constipation [8–12, 14–20]. The characteristics of all included studies are listed in Table 1.

### 3.3. Risk of Bias Assessment for the Included Studies

**Figure 2:**
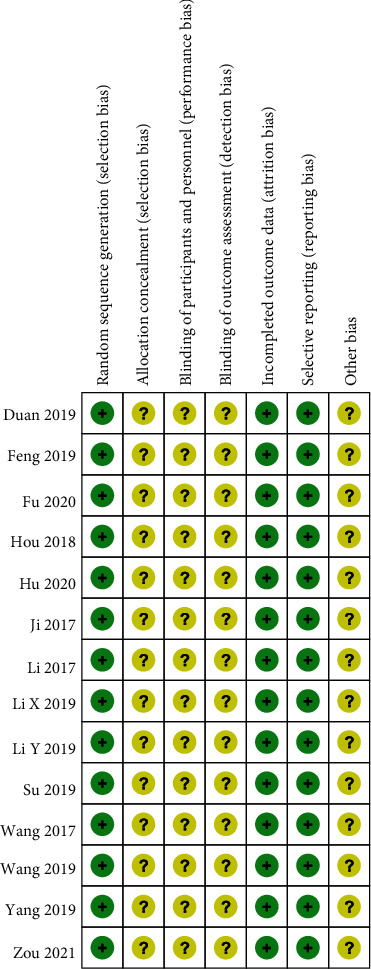
Risk of bias summary. +: low risk of bias; −: high risk of bias; ?: bias unclear.

Figure 2 shows the risk of bias assessment for the included 14 RCTs [7–20]. Random assignment was stated in all 14 studies, but none of them explicitly mentioned blinding and allocation concealment. Selective reporting or incomplete outcome data were also not found. Other biases could not be identified.

### 3.4. Results of the Meta-Analysis

#### 3.4.1. BMD

**Figure 3:**
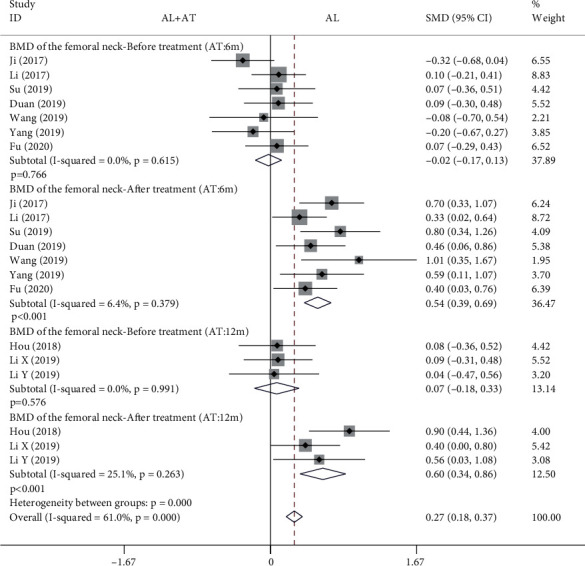
Forest plot showing the effect of alendronate combined with atorvastatin group compared to the alendronate alone group in BMD of the femoral neck (BMD: bone mineral density; AL: alendronate; AT: atorvastatin; SMD: standard mean difference).

Among the 14 included studies, there are a total of 10 studies with BMD as the primary outcome measurement [7, 8, 10, 11, 13–18]. The BMD is classified into 4 types depending on the measurement site, including femoral neck, femoral trochanter, forearm, and lumbar spine. Each BMD, in turn, was divided into 4 subgroups depending on the time of detection and the period of application of the intervention. The forest plot in Figure 3 shows the effect of alendronate combined with the atorvastatin group compared to the alendronate alone group in BMD of the femoral neck. Given that there are studies in which atorvastatin was given for 6 months and also for 12 months, it is discussed in subgroups. A total of 7 studies (688 patients) [7, 8, 11, 15–18] provided data for 6-month dosing cycles of atorvastatin and 3 studies (238 patients) [10, 13, 14] provided data for 12-month dosing cycles of atorvastatin. The fixed effects model was applied to this analytical process because *I*^2^ was less than 50%. According to the results of the pooled analysis, there was no statistically significant difference between the two groups before treatment (6-month atorvastatin: SMD = −0.02, 95% CI: [-0.17, 0.13], *P* = 0.766, *I*^2^ = 0%; 12-month atorvastatin: SMD = 0.07, 95% CI: [-0.18, 0.33], *P* = 0.576, *I*^2^ = 0%). However, according to the results of the pooled analysis, there were statistically significant differences between the two groups after treatment (6-month atorvastatin: SMD = 0.54, 95% CI: [0.39, 0.69], *P* < 0.001, *I*^2^ = 6.4%; 12-month atorvastatin: SMD = 0.6, 95% CI: [0.34, 0.86], *P* < 0.001, *I*^2^ = 25.1%).

**Figure 4:**
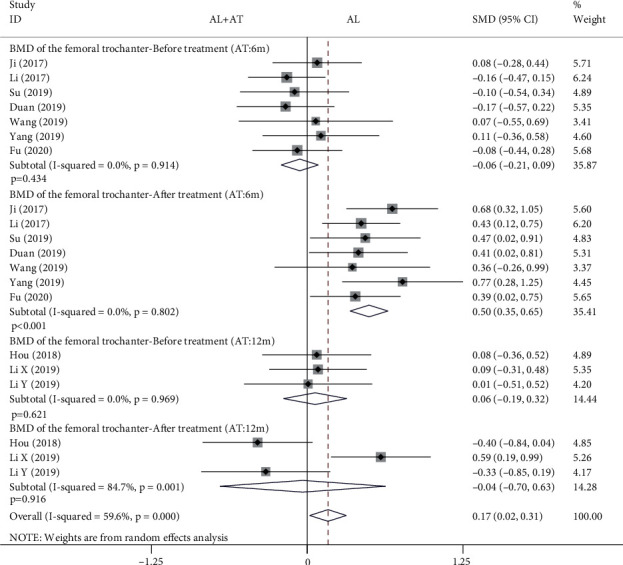
Forest plot showing the effect of alendronate combined with atorvastatin group compared to the alendronate alone group in BMD of the femoral trochanter (BMD: bone mineral density; AL: alendronate; AT: atorvastatin).

The forest plot in Figure 4 shows the effect of the alendronate combined with atorvastatin group compared to the alendronate alone group in BMD of the femoral trochanter. The random effects model was applied to this analytical process because *I*^2^ was greater than 50%. According to the results of the pooled analysis, there was no statistically significant difference between the two groups before treatment and a statistically significant difference after treatment when the atorvastatin dosing period was 6 months (before treatment: SMD = −0.06, 95% CI: [-0.21, 0.09], *P* = 0.434, *I*^2^ = 0%; after treatment: SMD = 0.5, 95% CI: [0.35, 0.65], *P* < 0.001, *I*^2^ = 0%). However, according to the results of the pooled analysis, there was no statistically significant difference between the two groups before and after treatment when the atorvastatin dosing period was 12 months (before treatment: SMD = 0.06, 95% CI: [-0.19, 0.32], *P* = 0.621, *I*^2^ = 0%; after treatment: SMD = −0.04, 95% CI: [-0.7, 0.63], *P* = 0.916, *I*^2^ = 84.7%).

**Figure 5:**
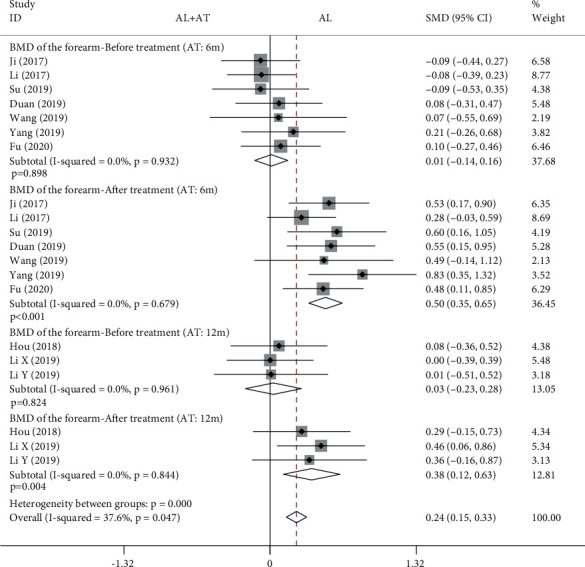
Forest plot showing the effect of alendronate combined with atorvastatin group compared to the alendronate alone group in BMD of the forearm (BMD: bone mineral density; AL: alendronate; AT: atorvastatin).

The forest plot in Figure 5 shows the effect of the alendronate combined with atorvastatin group compared to the alendronate alone group in BMD of the forearm. The fixed effects model was applied to this analytical process because *I*^2^ was less than 50%. According to the results of the pooled analysis, there was no statistically significant difference between the two groups before treatment (6-month atorvastatin: SMD = 0.01, 95% CI: [-0.14, 0.16], *P* = 0.898, *I*^2^ = 0%; 12-month atorvastatin: SMD = 0.03, 95% CI: [-0.23, 0.28], *P* = 0.824, *I*^2^ = 0%). However, according to the results of the pooled analysis, there were statistically significant differences between the two groups after treatment (6-month atorvastatin: SMD = 0.5, 95% CI: [0.35, 0.65], *P* < 0.001, *I*^2^ = 0%; 12-month atorvastatin: SMD = 0.38, 95% CI: [0.12, 0.63], *P* = 0.004, *I*^2^ = 0%).

**Figure 6:**
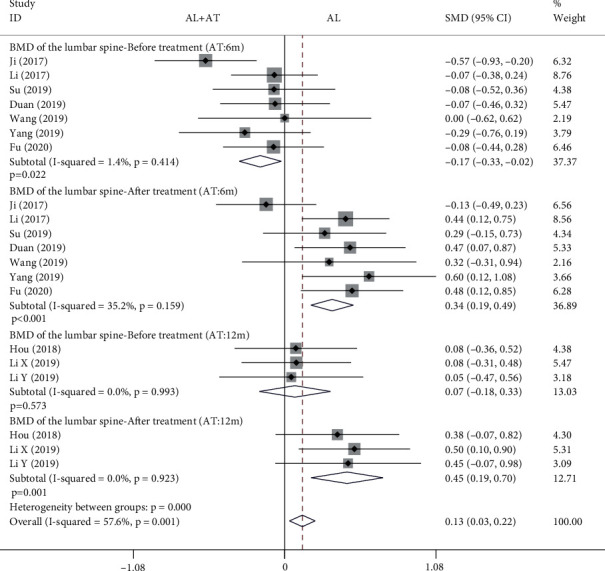
Forest plot showing the effect of alendronate combined with atorvastatin group compared to the alendronate alone group in BMD of the lumbar spine (BMD: bone mineral density; AL: alendronate; AT: atorvastatin).

The forest plot in Figure 6 shows the effect of the alendronate combined with atorvastatin group compared to the alendronate alone group in BMD of the lumbar spine. The fixed effects model was applied to this analytical process because *I*^2^ was less than 50%. According to the results of the pooled analysis, there was no statistically significant difference between the two groups before treatment and a statistically significant difference after treatment when the atorvastatin dosing period was 12 months (before treatment: SMD = 0.07, 95% CI: [-0.18, 0.33], *P* = 0.573, *I*^2^ = 0%; after treatment: SMD = 0.45, 95% CI: [0.19, 0.7], *P* = 0.001, *I*^2^ = 0%). However, according to the results of the pooled analysis, there were statistically significant differences between the two groups before and after treatment when the atorvastatin dosing period was 6 months (before treatment: SMD = −0.17, 95% CI: [-0.33, -0.02], *P* = 0.022, *I*^2^ = 1.4%; after treatment: SMD = 0.34, 95% CI: [0.19, 0.49], *P* < 0.001, *I*^2^ = 35.2%).

#### 3.4.2. Total Effective Rate

**Figure 7:**
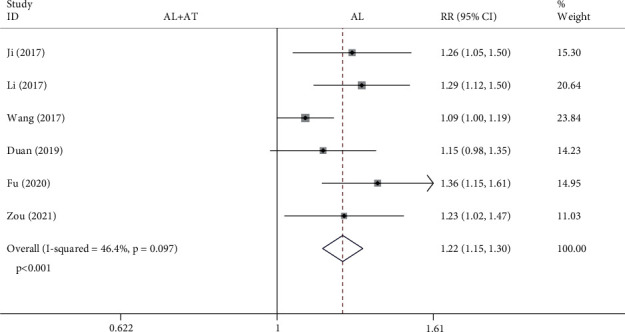
Forest plot showing the effect of alendronate combined with atorvastatin group compared to the alendronate alone group on total effective rate (AL: alendronate; AT: atorvastatin).

Among the 14 included studies, there are a total of 6 studies (728 patients) with the total effective rate as the secondary outcome measurement [7–9, 11, 18, 20]. The forest plot shown in Figure 7 shows the effect of the alendronate combined with atorvastatin group compared to the alendronate alone group on total effective rate. The fixed effects model was applied to this analytical process because *I*^2^ was less than 50%. According to the results of the pooled analysis, there was a statistically significant difference between the two groups (SMD = 1.22, 95% CI: [1.15, 1.3], *P* < 0.001, *I*^2^ = 46.4%).

#### 3.4.3. VAS

**Figure 8:**
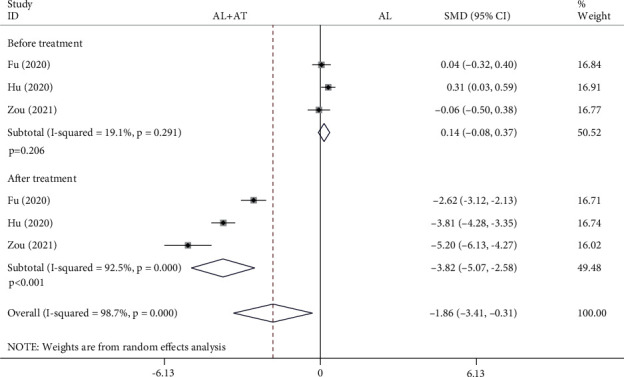
Forest plot showing the effect of alendronate combined with atorvastatin group compared to the alendronate alone group on VAS (AL: alendronate; AT: atorvastatin).

Among the 14 included studies, there are a total of 3 studies (398 patients) with VAS as the secondary outcome measurement [18–20]. The analysis was divided into two subgroups according to the time of detection. The forest plot shown in Figure 8 shows the effect of the alendronate combined with atorvastatin group compared to the alendronate alone group on VAS. The random effects model was applied to this analytical process because *I*^2^ was greater than 50%. According to the results of the pooled analysis, there was no statistically significant difference between the two groups before treatment and a statistically significant difference after treatment (before treatment: SMD = 0.14, 95% CI: [-0.08, 0.37], *P* = 0.206, *I*^2^ = 19.1%; after treatment: SMD = −3.82, 95% CI: [-5.07, -2.58], *P* < 0.001, *I*^2^ = 92.5%).

#### 3.4.4. OPG, BGP, and BAP

**Figure 9:**
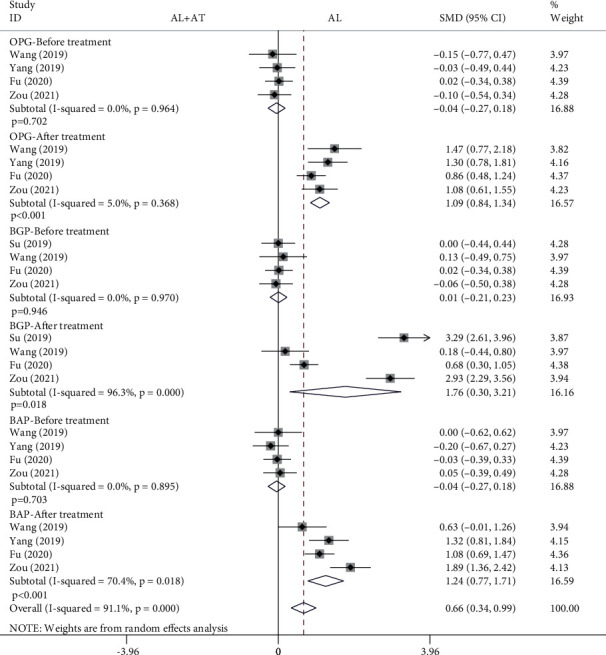
Forest plot showing the effect of alendronate combined with atorvastatin group compared to the alendronate alone group on OPG, BGP, and BAP (OPG: osteoprotegerin; BGP: bone Gla protein; BAP: bone alkaline phosphatase; AL: alendronate; AT: atorvastatin).

Among the 14 included studies, there are a total of 5 studies with OPG, BGP, and BAP as the secondary outcome measurements [15–18, 20]. The forest plot shown in Figure 9 shows the effect of the alendronate combined with atorvastatin group compared to the alendronate alone group on OPG, BGP, and BAP. A total of four studies (308 patients) provided OPG and BAP data [16–18, 20]; four studies (318 patients) provided BGP data [15, 16, 18, 20]. The random effects model was applied to this analytical process because *I*^2^ was greater than 50%. According to the results of the pooled analysis, there was no statistically significant difference between the two groups before treatment (OPG: SMD = −0.04, 95% CI: [-0.27, 0.18], *P* = 0.702, *I*^2^ = 0%; BGP: SMD = 0.01, 95% CI: [-0.21, 0.23], *P* = 0.946, *I*^2^ = 0%; BAP: SMD = −0.04, 95% CI: [-0.27, 0.18], *P* = 0.703, *I*^2^ = 0%). However, according to the results of the pooled analysis, there were statistically significant differences between the two groups after treatment (OPG: SMD = 1.09, 95% CI: [0.84, 1.34], *P* < 0.001, *I*^2^ = 5%; BGP: SMD = 1.76, 95% CI: [0.3, 3.21], *P* = 0.018, *I*^2^ = 96.3%; BAP: SMD = 1.24, 95% CI: [0.77, 1.71], *P* < 0.001, *I*^2^ = 70.4%).

#### 3.4.5. Blood P and Ca

**Figure 10:**
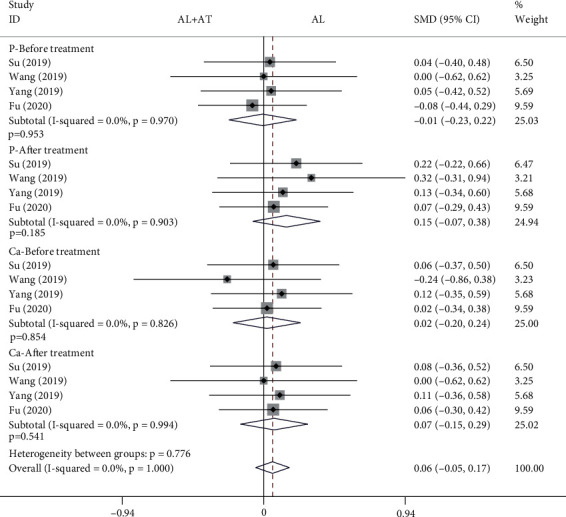
Forest plot showing the effect of alendronate combined with atorvastatin group compared to the alendronate alone group on blood P and Ca (AL: alendronate; AT: atorvastatin).

Among the 14 included studies, there are a total of 4 studies (308 patients) with blood P and Ca as the secondary outcome measurements [15–18]. The forest plot shown in Figure 10 shows the effect of the alendronate combined with atorvastatin group compared to the alendronate alone group on blood P and Ca. The fixed effects model was applied to this analytical process because *I*^2^ was less than 50%. According to the results of the pooled analysis, there was no statistically significant difference between the two groups before and after treatment on blood P and Ca (blood P-before treatment: SMD = −0.01, 95% CI: [-0.23, 0.22], *P* = 0.953, *I*^2^ = 0%; blood P-after treatment: SMD = 0.15, 95% CI: [-0.07, 0.38], *P* = 0.185, *I*^2^ = 0%; blood Ca-before treatment: SMD = 0.02, 95% CI: [-0.2, 0.24], *P* = 0.854, *I*^2^ = 0%; blood Ca-after treatment: SMD = 0.07, 95% CI: [-0.15, 0.29], *P* = 0.541, *I*^2^ = 0%).

#### 3.4.6. Adverse Events

**Figure 11:**
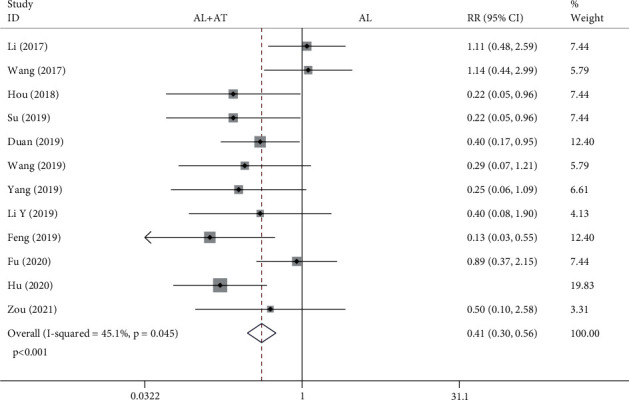
Forest plot showing the results regarding adverse events of alendronate combined with atorvastatin group compared to the alendronate alone group (AL: alendronate; AT: atorvastatin).

Twelve studies reported adverse events, including headache, abdominal pain, nausea, vomiting, and constipation [8–12, 14–20]. The forest plot shown in Figure 11 shows the results regarding adverse events of the alendronate combined with atorvastatin group compared to the alendronate alone group. The fixed effects model was applied to this analytical process because *I*^2^ was less than 50%. According to the results of the pooled analysis, there was a statistically significant difference between the two groups on adverse events (RR = 0.41, 95% CI: [0.3, 0.56], *P* < 0.001, *I*^2^ = 45.1%).

### 3.5. Publication Bias

Begg's funnel plot and Egger's test are now commonly used in meta-analyses to assess publication bias, usually for at least 10 studies [23]. Since *P* < 0.05 for Begg's test and Egger's test results, this suggests a possible publication bias for the included studies of total effective rate (Egger's test: *P* = 0.048), adverse events (Egger's test: *P* = 0.043), and OPG, BGP, and BAP (Begg's test: *P* = 0.003, Egger's test: *P* = 0.04). No bias was published for other outcome measurements as the results of Begg's test and Egger's test *P* > 0.05.

### 3.6. Sensitivity Analysis

When the results are heterogeneous, sensitivity analysis is usually performed in a meta-analysis to assess the stability of the results of the pooled literature analysis. We used sensitivity analysis to remove all outcome measurements from all included literature one by one, and the above results did not change significantly, which implies the robustness of the results.

## 4. Discussion

This meta-analysis explored the effect of the alendronate combined with atorvastatin group compared to the alendronate alone group in patients with osteoporosis in diabetes mellitus. Diabetes is a lifelong endocrine disease, and osteoporosis is a serious complication of diabetes [3]. The high fasting blood glucose level caused by the impairment of insulin metabolism in the body prompts hyperparathyroidism and then hyperalgesia, resulting in the inability to effectively convert vitamin D to active vitamin D. This in turn triggers abnormal bone metabolism in the body, leading to a decrease in bone content and osteoporosis. Clinical symptoms include prolonged pain and dysfunction of the bones, easy to fracture, and not easy to heal, which seriously affect the quality of life and safety of patients [2]. Therefore, more attention should be paid to the treatment and prevention of osteoporosis, and it is crucial to find effective treatment methods. Alendronate is an aminobisphosphonate that acts as a potent inhibitor of bone resorption [4]. A clinical study showed that 70 mg of alendronate per week was effective in improving BMD and reducing bone loss in patients with proximal femur osteoporosis [4]. As a statin drug, atorvastatin has been widely used to lower cholesterol levels [5]. It has been reported that statins have a variety of effects, such as antioxidant properties, inhibition of inflammation, and bone metabolism. A nationwide population-based cohort study suggests that high-potency statins (atorvastatin and rosuvastatin) and moderate-potency statin (simvastatin) appear to have a potential protective effect against osteoporosis [5]. Some of the available studies have reported that the combination of alendronate and atorvastatin is more effective than alendronate alone in the treatment of osteoporosis in diabetes mellitus [7–20]. Therefore, we performed this meta-analysis to pool related studies and to assess the effectiveness of the combination group and the alendronate alone group.

A total of 14 articles that met the inclusion criteria were included in this meta-analysis [7–20]. The experimental group in all studies was alendronate in combination with atorvastatin, while the control group was alendronate alone, and the patients were diabetic with osteoporosis. BMD measurement was divided into 4 types according to the measurement site, including the femoral neck, femoral trochanter, forearm, and lumbar spine. Based on the results of the pooled analysis, it was concluded that BMD of the femoral neck was higher with alendronate combined with atorvastatin than with alendronate alone, regardless of whether the cycle of atorvastatin application was 6 (before treatment: SMD = −0.02, 95% CI: [-0.17, 0.13], *P* = 0.766, *I*^2^ = 0%; after treatment: SMD = 0.54, 95% CI: [0.39, 0.69], *P* < 0.001, *I*^2^ = 6.4%) or 12 months (before treatment: SMD = 0.07, 95% CI: [-0.18, 0.33], *P* = 0.576, *I*^2^ = 0%; after treatment: SMD = 0.6, 95% CI: [0.34, 0.86], *P* < 0.001, *I*^2^ = 25.1%). When the site of measurement was the femoral trochanter, BMD was higher with alendronate combined with atorvastatin (6 months) than with alendronate alone (before treatment: SMD = −0.06, 95% CI: [-0.21, 0.09], *P* = 0.434, *I*^2^ = 0%; after treatment: SMD = 0.5, 95% CI: [0.35, 0.65], *P* < 0.001, *I*^2^ = 0%), while there was no significant difference between the two groups at 12 months (before treatment: SMD = 0.06, 95% CI: [-0.19, 0.32], *P* = 0.621, *I*^2^ = 0%; after treatment: SMD = −0.04, 95% CI: [-0.7, 0.63], *P* = 0.916, *I*^2^ = 84.7%). The results for the BMD of the forearm and femoral neck are consistent. When the measurement site was the lumbar spine and the period of atorvastatin application was 6 months, there were statistically significant differences in BMD between the two groups before treatment and after treatment, showing that BMD was higher with alendronate combined with atorvastatin than with alendronate alone (before treatment: SMD = −0.17, 95% CI: [-0.33, -0.02], *P* = 0.022, *I*^2^ = 1.4%; after treatment: SMD = 0.34, 95% CI: [0.19, 0.49], *P* < 0.001, *I*^2^ = 35.2%); it was also higher in the combination group when atorvastatin was applied for 12 months (before treatment: SMD = 0.07, 95% CI: [-0.18, 0.33], *P* = 0.573, *I*^2^ = 0%; after treatment: SMD = 0.45, 95% CI: [0.19, 0.7], *P* = 0.001, *I*^2^ = 0%). A pooled analysis of the total effective rate showed that alendronate combined with atorvastatin was more effective than alendronate alone in the treatment of osteoporosis in diabetes mellitus (SMD = 1.22, 95% CI: [1.15, 1.3], *P* < 0.001, *I*^2^ = 46.4%). The VAS results indicated that alendronate combined with atorvastatin was more effective in relieving pain than alendronate alone (before treatment: SMD = 0.14, 95% CI: [-0.08, 0.37], *P* = 0.206, *I*^2^ = 19.1%; after treatment: SMD = −3.82, 95% CI: [-5.07, -2.58], *P* < 0.001, *I*^2^ = 92.5%). Serological findings showed that alendronate combined with atorvastatin had higher OPG, BGP, and BAP than alendronate alone (before treatment: OPG: SMD = −0.04, 95% CI: [-0.27, 0.18], *P* = 0.702, *I*^2^ = 0%; BGP: SMD = 0.01, 95% CI: [-0.21, 0.23], *P* = 0.946, *I*^2^ = 0%; BAP: SMD = −0.04, 95% CI: [-0.27, 0.18], *P* = 0.703, *I*^2^ = 0%) (after treatment: OPG: SMD = 1.09, 95% CI: [0.84, 1.34], *P* < 0.001, *I*^2^ = 5%; BGP: SMD = 1.76, 95% CI: [0.3, 3.21], *P* = 0.018, *I*^2^ = 96.3%; BAP: SMD = 1.24, 95% CI: [0.77, 1.71], *P* < 0.001, *I*^2^ = 70.4%), while there were no significant differences in serum P and Ca between the two groups (blood P-before treatment: SMD = −0.01, 95% CI: [-0.23, 0.22], *P* = 0.953, *I*^2^ = 0%; blood P-after treatment: SMD = 0.15, 95% CI: [-0.07, 0.38], *P* = 0.185, *I*^2^ = 0%; blood Ca-before treatment: SMD = 0.02, 95% CI: [-0.2, 0.24], *P* = 0.854, *I*^2^ = 0%; blood Ca-after treatment: SMD = 0.07, 95% CI: [-0.15, 0.29], *P* = 0.541, *I*^2^ = 0%). A pooled analysis of adverse events showed a higher incidence of adverse events in the alendronate alone group (RR = 0.41, 95% CI: [0.3, 0.56], *P* < 0.001, *I*^2^ = 45.1%). These results suggest that alendronate combined with atorvastatin is more effective than alendronate alone in treating osteoporosis in diabetes mellitus, with higher BMD, fewer adverse events, and more significant pain relief.

### 4.1. Limitations

This meta-analysis has some limitations due to the number and quality of the included studies. First, some studies lacked details such as blinding and allocation concealment. Second, the heterogeneity of some results was high. The heterogeneity may be at least partially due to the difference of BMD measurement. It is known that many methods can be used to measure BMD, such as micro-CT, dual-energy X-ray absorptiometry, and ultrasound. Dual-energy X-ray absorptiometry is much less sensitive in recording changes than a CT scan of the bone. Different methods used in different studies may lead to the possible heterogeneous results. Finally, the included studies generally lacked the timing of testing for outcome measurements.

## 5. Conclusion

This is a meta-analysis to evaluate the effectiveness of alendronate combined with atorvastatin compared with alendronate alone in the treatment of osteoporosis in diabetes mellitus. Our results showed that alendronate combined with atorvastatin is more effective than alendronate alone, with higher BMD, OPG, BGP, and BAP; more significant pain relief; and fewer adverse events. Due to the limited number and quality of relevant studies, more high-quality RCTs are still needed in the future to complement the existing findings.

## Figures and Tables

**Table 1:** Characteristic of all studies included in the meta-analysis.

Study	Country	Study type	Mean age (years) E : C	No. of patients E : C	Mean BMI (kg/m^2^) E : C	Experimental group	Experimental dose	Control group	Control dose	Outcome measures
Ji et al., 2017	China	RCT	64.6/64.2	60/60	25/23	AL+AT	10 mg/d/6 m + 10 mg/d/6 m	AL	10 mg/d/6 m	A, G
Su et al., 2019	China	RCT	64/64	40/40	NP	AL+AT	10 mg/d/6 m + 10 mg/d/6 m	AL	10 mg/d/6 m	A, D, F, H
Duan et al., 2019	China	RCT	67.58/68.39	50/50	NP	AL+AT	10 mg/d/6 m + 10 mg/d/6 m	AL	10 mg/d/6 m	A, G, H
Fu et al., 2020	China	RCT	65.7/65.9	59/59	24.95/25.03	AL+AT	10 mg/d/6 m + 10 mg/d/6 m	AL	10 mg/d/6 m	A, B, C, D, E, F, G, H
Zou et al., 2021	China	RCT	62.45/62.48	40/40	23.55/23.51	AL+AT	10 mg/d/6 m + 10 mg/d/6 m	AL	10 mg/d/6 m	B, C, D, E, G, H
Li et al., 2017	China	RCT	65.5/66.5	90/90	24.58/25.5	AL+AT	70 mg/w/6 m + 10 mg/d/6 m	AL	70 mg/w/6 m	A, G, H
Wang et al., 2017	China	RCT	68.7/67.2	75/75	NP	AL+AT	70 mg/w/6 m + 10 mg/d/6 m	AL	70 mg/w/6 m	G, H
Wang et al., 2019	China	RCT	64.2/63.3	20/20	NP	AL+AT	70 mg/w/6 m + 10 mg/d/6 m	AL	70 mg/w/6 m	A, C, D, E, F, H
Yang et al., 2019	China	RCT	66.27/66.43	35/35	23.04/23.25	AL+AT	70 mg/w/6 m + 10 mg/d/6 m	AL	70 mg/w/6 m	A, C, E, F, H
Hu et al., 2020	China	RCT	58.23/57.89	100/100	NP	AL+AT	70 mg/w/6 m + 10 mg/d/6 m	AL	70 mg/w/6 m	B, H
Hou et al., 2018	China	RCT	64.5/62.5	40/40	NP	AL+AT	70 mg/w/6 m + 10 mg/d/12 m	AL	70 mg/w/6 m	A, H
Li X et al., 2019	China	RCT	65.09/65.43	50/50	NP	AL+AT	10 mg/w/6 m + 10 mg/d/12 m	AL	10 mg/d/6 m	A, B
Li Y et al., 2019	China	RCT	68.4/67.5	29/29	NP	AL+AT	70 mg/w/6 m + 10 mg/d/12 m	AL	70 mg/w/6 m	A, H
Feng et al., 2019	China	RCT	58.93/61.9	50/50	NP	AL+AT	70 mg/w/6 m + 10 mg/d/12 m	AL	70 mg/w/6 m	H

E: experimental group; C: control group; RCT: randomized controlled trial; BMI: body mass index; AL: alendronate; AT: atorvastatin; d: day; w: week; m: month; NP: not provided. Outcome measure: A: BMD (femoral neck, femoral trochanter, forearm, lumbar spine); B: VAS; C: OPG; D: BGP; E: BAP; F: blood P and Ca; G: total effective rate; H: adverse events.

## Data Availability

The data supporting this meta-analysis is from previously reported studies and datasets, which have been cited.
